# Efficacy of Two Irrigants Used with Self-Adjusting File System on Smear Layer: A Scanning Electron Microscopy Study

**DOI:** 10.1155/2014/289164

**Published:** 2014-09-23

**Authors:** Özgür Genç Şen, Sadullah Kaya, Özgür Er, Tayfun Alaçam

**Affiliations:** ^1^Department of Endodontics, Faculty of Dentistry, Yüzüncü Yıl University, 65080 Van, Turkey; ^2^Department of Endodontics, Faculty of Dentistry, Dicle University, 21280 Diyarbakır, Turkey; ^3^Department of Endodontics, Faculty of Dentistry, Erciyes University, 38039 Kayseri, Turkey; ^4^Department of Endodontics, Faculty of Dentistry, Gazi University, 06510 Ankara, Turkey

## Abstract

Mechanical instrumentation of root canals produces a smear layer that adversely affects the root canal seal. The aim of this study was to evaluate efficacy of MTAD and citric acid solutions used with self-adjusting file (SAF) system on smear layer. Twenty-three single-rooted human teeth were used for the study. Canals were instrumented manually up to a number 20 K file size. SAF was used to prepare the root canals. The following groups were studied: Group 1: MTAD + 5.25% NaOCl, Group 2: 20% citric acid + 5.25% NaOCl, and Group 3: Control (5.25% NaOCl). All roots were split longitudinally and subjected to scanning electron microscopy. The presence of smear layer in the coronal, middle, and apical thirds was evaluated using a five-score evaluation system. Kruskal-Wallis and Mann-Whitney *U* tests were used for statistical analysis. In the coronal third, Group 2 exhibited the best results and was statistically different froms the other groups (*P* < 0.05). There was not a significant difference among the three thirds of groups according to in-group comparisons (*P* > 0.05). The solutions used in Group 1 and 2 could effectively remove smear layer in most of the specimens. However, citric acid was more effective than MTAD in the three thirds of the canal.

## 1. Introduction

Adequate debridement of the root canal plays an important role in the success of root canal treatment [1]. Mechanical instrumentation of root canals produces a smear layer comprising organic and inorganic substances, such as dentin particles, necrotic debris, microorganisms, and odontoblastic processes [2]. Despite the controversies regarding the smear layer [3, 4], the general consensus is that the smear layer adversely affects the root canal seal [5, 6]. Several chemicals have therefore been investigated as irrigants to remove the smear layer. The most commonly used irrigation solutions are chelating agents and acids. The effectiveness of citric acid for removal of the smear layer was demonstrated in the 1970s [7, 8]. Torabinejad et al. [9] reported an irrigation solution comprising a mixture of 3% doxycycline, 4.25% citric acid, and 0.5% polysorbate 80 detergent, named MTAD. The quantity of smear layer removed by an irrigation solution is related to its pH and the exposure time. Traditionally, irrigants are delivered by a large syringe and needle to facilitate their insertion [10]. But they can progress only 1 mm beyond the tip of the needle [11]. Therefore, several mechanical devices have been developed to improve the penetration and effectiveness of irrigants. Some of these irrigation techniques include manual irrigation with needles and cannulas and the use of machine-assisted agitation systems with sonic and ultrasonic energy sources [12].

Self-adjusting file (SAF) was introduced in 2010 [13] and claimed to be successful in difficult-to-clean parts of the root canal with continuous flow of the irrigant. The aim of this study was to evaluate efficacy of MTAD and citric acid solutions used with SAF system on smear layer.

## 2. Materials and Methods

Twenty-three freshly extracted single-rooted human teeth with a straight canal, stored in saline solution, were used. Radiographs of the teeth were taken in the buccolingual and mesiodistal projections to analyze the shape of the root canals and to detect possible anatomic variations.

The coronal parts of the teeth were cut with a high-speed diamond bur to standardize the root lengths and to provide direct access to the root canals. Number 15 K files (Dentsply-Maillefer, Ballaigues, Switzerland) were introduced further into the root canals until their tips were visible at the apical foramen. The working length was determined as 1 mm shorter than this length. The canals were instrumented manually up to number 20 K file. 5 mL, 5.25% sodium hypochlorite (NaOCl) was used for irrigation between the instruments.

A SAF file (ReDent-Nova, Israel) was used to prepare the root canals as described by Metzger et al. [13]. Irrigation was performed continuously during the instrumentation using a special irrigation apparatus (VATEA Irrigation Device, ReDent-Nova, Israel). This apparatus has two separate irrigant reservoirs connected to a hollow SAF file. Continuous irrigation was applied at a flow rate of 5 mL/min. The SAF file instrumentation with irrigation was performed for a total of 4 minutes in each root canal:

 Group 1: 10 roots were used. 5.25% NaOCl was used for 3 minutes (at a flow rate of 5 mL/min, 15 mL in total) and then 5 mL MTAD was used for 1 minute;
 Group 2: 10 roots were used. 5.25% NaOCl was used for 3 minutes (at a flow rate of 5 mL/min, 15 mL in total) and then 5 mL 20% citric acid was used for 1 minute;
 Group 3 (Control group): 3 roots were used. 5.25% NaOCl solution were used for 4 minutes (at a flow rate of 5 mL/min, 20 mL in total).

Finally, all roots were irrigated with 5 mL distilled water, then dried with sterile paper points, and left to dry at a room temperature for 24 hours.

All roots were grooved longitudinally on the external surface with a diamond disc in the buccolingual plane, avoiding penetration of the root canals. The roots were separated into two halves with a chisel. The specimens were fixed on metal holders and coated with gold and viewed with FEI Quanta 400F field-emission scanning electron microscope (FEI Company, Hillsboro, OR, USA). The most accessible areas in each third were selected and photomicrographed. The smear layer was evaluated from images at 2000x magnification based on the scale of Hülsmann et al. [14]: score 1, no smear layer and all dentinal tubules were open; score 2, a small amount of smear layer and some dentinal tubules were open; score 3, homogeneous smear layer covering the root canal wall and only a few dentinal tubules were open; score 4, complete root canal wall covered by a homogeneous smear layer and no open dentinal tubules; and score 5, heavy homogeneous smear layer covering the complete root canal. Scores 1 and 2 represent “clean canal wall.” Scores 3, 4, and 5 represent “smear layer present.” The Kruskal-Wallis test was used for statistical evaluation and Mann-Whitney *U* test was used for multiple comparisons.

## 3. Results

**Figure 1:**
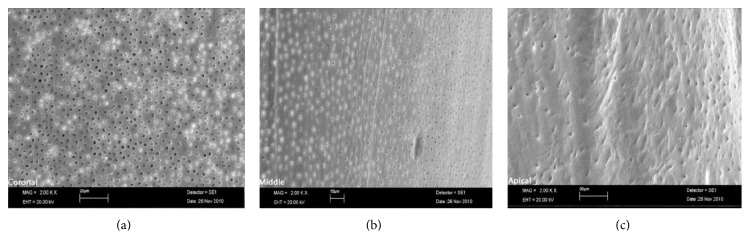
SEM micrograph of Group 1: (a) coronal third, (b) middle third, and (c) apical third.

**Figure 2:**
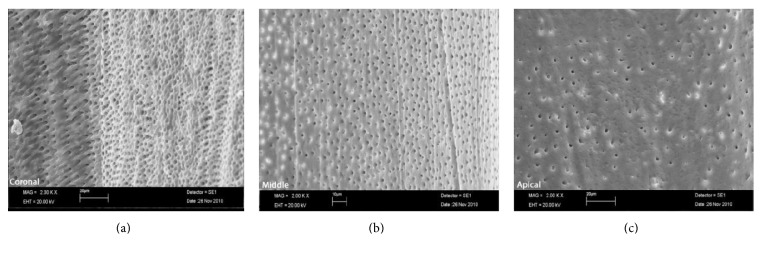
SEM micrograph of Group 2: (a) coronal third, (b) middle third, and (c) apical third.

**Figure 3:**
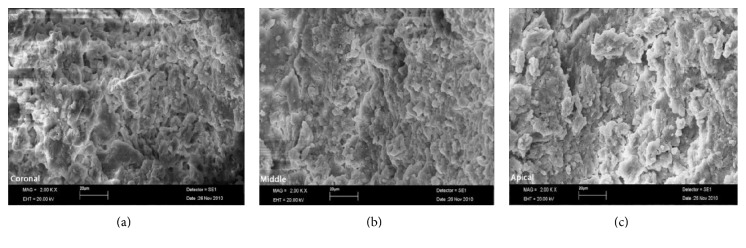
SEM micrograph of Group 3: (a) coronal third, (b) middle third, and (c) apical third.

The SAF, operated with MTAD-NaOCl and citric acid-NaOCl, resulted in clean canals and most of the specimens revealed scores 1 and 2 (Figures 1 and 2). The cleaning rates of Groups 1 and 2 are shown in Tables 1 and 2. Control group exhibited heavy smear layer covering the root canal walls (Figure 3).

### 3.1. Comparison of Different Thirds within Each Group (In-Group Comparisons of Thirds)

No significant difference was found statistically in the smear layer on the dentine wall among the coronal, middle, and apical thirds in Groups 1, 2, and 3 based on comparisons within each group (Group 1, *P* = 0.378; Group 2, *P* = 0.065; Group 3, *P* = 1.00) (Kruskal-Wallis test).

### 3.2. Comparison of the Same Thirds between Groups (Intergroup Comparisons of Thirds)

Mean scores and statistical equivalence related to the thirds of teeth in the three groups are shown in Table 3. In the coronal third, there was a statistically significant difference among the three groups (Groups 1-2, *P* = 0.005; Groups 1–3, *P* = 0.007; Groups 2-3, *P* = 0.005). Group 3 (Control) was statistically different from the other groups also in the middle (Groups 1–3, *P* = 0.009; Groups 2-3, *P* = 0.007) and apical thirds (Groups 1–3, *P* = 0.009; Groups 2-3, *P* = 0.009) (Mann-Whitney *U* test).

## 4. Discussion

Similar to hand and rotary instrumentation, the SAF system produces a smear layer when using NaOCl alone, but when alternating the application of MTAD and 5.25% NaOCl and 20% citric acid and 5.25% NaOCl, the canals were rendered virtually free of debris and smear layer, with the most pronounced benefit realized in the apical third of the root canal, as confirmed by the present study.

In recent years, numerous researchers [15–19] studied SAF system using different evaluation methods and reported successful results as our study in shaping and irrigating root canals. However, as a result of a microbiological and scanning electron microscopy study, Paranjpe et al. [20] found out insufficient apical preparation and irrigation when using the SAF system. This result could be explained by differences in the sample and the testing methods.

In the past, different concentrations of citric acid were used to remove the smear layer [21, 22]. di Lenarda et al. [23] reported no or negligible difference in smear layer removal with citric acid and EDTA, the most common chelating agent. In a study of Mancini et al. [24], the efficacy of 42% citric acid, MTAD, and 17% EDTA was tested on removing the smear layer. The irrigation solutions were delivered via a nickel-titanium needle (Stropko NiTi Flexi-Tip; SybronEndo, Orange, CA), which penetrated within 1 to 2 mm of the working length. In direct contradiction with our study, none of these three solutions removed the smear layer in the apical third of the root canal. Numerous investigations [25–28] revealed that extended exposure to acids results in excessive demineralization. Therefore, 4 min of 20% citric acid application instead of 40% was used with a final flush of saline in the current study. Although we used a lower concentration of citric acid (20%), the smear layer was successfully removed in the majority of our specimens. This success can be attributed to the continuous irrigation and vibration action of the SAF system. In the study of Metzger et al. [29], the use of the SAF system and irrigation with EDTA and NaOCl resulted in smear layer-free canal walls in the apical third of 65% of the specimens. The VATEA peristaltic pump used in the SAF system delivers a continuous flow of irrigant, which enters the canal through the hollow file. According to the manufacturer, the motion of the file agitates the irrigant to such an extent that it effectively reaches the apical part of the canal with sonic activation. We thought that continuous replacement of irrigant could also explain the excellent cleaning efficiency observed in this study.

Akhlaghi et al. [30] reported successful cleaning in apical portion of canals irrigated with MTAD. In their study, MTAD irrigation was achieved using a 28-gauge needle placed into the canal space 1 to 1.5 mm short of the working length and a cotton-wrapped barbed broach was placed to the end of the working length and left there for a few minutes. The canal was then reirrigated with MTAD. These researchers reported smear-free dentine walls in the apical third of the canals. This success might be due to the direct contact of the fresh solution and replacement of the older solution by the cotton wrapped barbed broach. Similarly, direct contact of fresh irrigant with dentine walls might facilitate the SAF system irrigation with MTAD in our study. As Zehnder [31] emphasized, optimal cleaning requires direct contact of the irrigant with the root canal surface.

In a study by Tay et al. [32], BioPure MTAD and EDTA were applied as a final irrigant, and to increase contact and penetration, a gutta-percha point was used to agitate the solution. The authors reported that the smear layer was successfully removed using this technique, regardless of the irrigant. In the present study, the irrigants were activated by the SAF action. Metzger et al. [29] claimed that the SAF file has a scrubbing action on the canals, which clearly results in a very clean surface even in the unreachable parts of the canal by activation of the irrigant in the apical third of the canal. In a recent study of Melo Ribeiro et al. [33], oval SAF was used with continuous NaOCl irrigation on oval-shaped root canals. These researchers reported that the percentage of remaining debris and uninstrumented canal perimeter was significantly lower in the SAF group than in the rotary group. Previously, De-Deus et al. [18] explained this result by the ability of SAF instrument to adapt itself to the cross-section of the canal and the mechanical debridement efficacy of its continuous irrigation system.

Although there is general agreement regarding the necessity of removing the smear layer, the optimal irrigation solution and removal technique remain under debate.The present findings revealed the effectiveness of the SAF system with two different irrigation solutions, suggesting that this methodology may be a useful alternative to conventional methods. Further studies are required to determine the most effective parameters.

## 5. Conclusion

In the limitations of this study, it can be concluded that using the SAF system and continuous irrigation action with EDTA and MTAD solutions could overcome the difficulty of removing smear layer even in hard-to-reach regions of the root canal.

## Figures and Tables

**Table 1:** Hülsmann scores given with the number of specimens that belong to each score and the percent of cleaning for Group 1 (MTAD + NaOCl).

	Group 1 coronal third					Group 1 middle third					Group 1 apical third				
	Clean		Smear layer present			Clean		Smear layer present			Clean		Smear layer present		
Score	1	2	3	4	5	1	2	3	4	5	1	2	3	4	5
*n*	1	6	3	—	—	4	3	3	—	—	1	5	2	2	—

Total	70%		30%			70%		30%			60/%		40%		

*n*: number of specimens.

**Table 2:** Hülsmann scores given with number of specimens that belong to each score and percent of cleaning for Group 2 (citric acid + NaOCl).

	Group 2 coronal third					Group 2 middle third					Group 2 apical third				
	Clean		Smear layer present			Clean		Smear layer present			Clean		Smear layer present		
Score	1	2	3	4	5	1	2	3	4	5	1	2	3	4	5
*n*	7	3	—	—	—	6	1	3	—	—	2	5	2	1	—

Total	100%		0%			70%		30%			70%		30%		

*n*: number of specimens.

**Table 3:** Mean of smear scores related to the thirds of teeth in Groups 1, 2, and 3 (intergroup comparisons of thirds).

	Coronal	Middle	Apical
Group 1 (MTAD + NaOCl)	2.2	1.9∗	2.5∗∗
Group 2 (citric acid + NaOCl)	1.3	1.7∗	2.2∗∗
Group 3 (NaOCl)	5	5	5

∗ and ∗∗ show the statistical equivalence.
